# Strategies for improving compliance with removable retention appliances: A qualitative study

**DOI:** 10.1177/14653125261450635

**Published:** 2026-05-28

**Authors:** Ingalill Feldmann, Anke Krämer, Mats Sjöström, Christina Storm Mienna

**Affiliations:** 1 Centre for Research and Development, Uppsala University/Region Gävleborg, Gävle, Sweden; 2 Orthodontic Clinic, Public Dental Health, Region Gävleborg, Gävle, Sweden; 3 Department of Odontology, Division of Oral and Maxillofacial Surgery, Umeå University, Umeå, Sweden; 4 Department of Odontology, Orofacial pain and Jaw function, Umeå University, Sweden; 5 Várdduo – Centre for Sámi Research, Umeå University, Sweden

**Keywords:** orthodontics, retention, compliance, adherence, qualitative, Grounded Theory

## Abstract

**Objective:**

To explore experiences affecting patients’ compliance with removable retention appliances, and to investigate strategies for improving adherence to wear recommendations from the profession.

**Design:**

A qualitative study designed alongside a randomised controlled trial regarding post-treatment stability and patients’ perceptions.

**Setting:**

Orthodontic Clinic, Public Dental Health Service, Region Gävleborg, Gävle, Sweden.

**Participants:**

A total of 15 purposively selected participants (age range = 25–32 years) with a recommendation of long-term retainer wear.

**Methods:**

In-depth interviews were performed, using a pre-tested topic guide, and analysed according to the qualitative method of Grounded Theory.

**Results:**

Compliance failures were preceded by one or more triggers that emerged after a couple of weeks or after several years of retention. A core category was identified and labelled ‘Pragmatic actions for improved adherence’, which summarises the decisions to either discontinue or continue to follow current retention protocol as being based on pragmatic patient-centred considerations. Data analysis revealed three interacting categories for improving compliance from both the patient and the clinician perspectives: Individual factors, Support, Self-motivation and Routines.

**Conclusions:**

To be successful, strategies to enhance compliance with removable retainers must originate from the individual patient and their life situation. Long-term support from both family and professionals, as well as good routines, are considered crucial for this process.

## Plain language summary

Strategies to increase compliance with stabilisation appliances after orthodontic treatment: An interview study

**Why was the study done?** After orthodontic treatment, teeth need to be kept in place so they do not move back. Many patients get removable retainers that must be worn for a long time. However, it can be hard to follow these instructions, and we know little about why some patients stop using their retainers.

**What did the researchers do?** We interviewed 15 adults who had used removable retainers for several years. The interviews were analysed using a qualitative method (Grounded Theory) to capture their experiences and strategies for following the recommendations.

**What did the researchers find?** The choice to keep wearing or stop wearing the retainers often depended on practical issues. Three main factors influencing adherence were identified: (1) individual factors such as personality, health and life situation; (2) support from family and dental professionals; and (3) self-motivation and routines, such as reminders and habits. Pain, discomfort and changes in life circumstances were common reasons for stopping retainer use.

**What do the findings mean?** To improve adherence, strategies must be adapted to each patient’s life situation. Long-term support from both family and dental professionals, along with clear routines and reminders, are important. This can lower the risk of teeth shifting back and reduce the need for new treatment.

## Introduction

There is consensus among orthodontists that teeth need to be stabilised after orthodontic treatment and patients are therefore provided with fixed or/and removable retainers for shorter or longer periods of time when active treatment is finished (Joondeph et al., 2017; Proffit, 2013). Fixed retainers are frequently used in both the maxilla and mandible (Al-Moghrabi et al., 2021) although they have clear short-comings such as higher risk for breakage (7%—50% of all bonded retainers), plaque and calculus accumulations, and risk of periodontal health issues (Aye et al., 2021; Jedliński et al., 2021; Pandis et al., 2007). Removable retainers can appear in different designs, for example the Hawley retainer and the vacuum-formed retainer (VFR), but with the common feature of being completely dependent on patient compliance (Al-Moghrabi et al., 2021). Removable retainer wear often involves periodic use during the daytime with a higher degree of discomfort, speech problems and occasional embarrassment for the patient, which can lead to compromised compliance (Forde et al., 2018; Frawley et al., 2022; Lindauer and Shoff, 1998). There is a geographical variation in the use of different retention protocols, as well as individual variation depending on the orthodontist’s experience (Hamran et al., 2022; Singh et al., 2009; Valiathan and Hughes, 2010). The current trend is towards increasingly prolonged retention phases, with some recommendations advocating lifelong retention (Singh et al., 2009; Valiathan and Hughes, 2010). From this perspective, the techniques used for retention become extremely important. Despite this, the latest Cochrane review concluded that definitive evidence supporting an ideal retention strategy is still lacking (Martin et al., 2023).

Research on orthodontic retention focuses mainly on treatment stability and post-treatment changes measured on dental casts using quantitative research methods. Several studies have investigated effectiveness of different fixed and removable retainers to identify the optimal retention strategy (Forde et al., 2018; Krämer et al., 2020; O’Rourke et al., 2016). The results reveal that relapse mainly occurs during the first 12 months of retention with both fixed and removable retainers. However, the results from studies with longer follow-up times show that post-treatment changes can still be expected after 4–5 years and that relapse occurs more frequently with removable retainers (Al-Moghrabi et al., 2018; Edman Tynelius et al., 2015; Krämer et al., 2023). Thus, retention appliances must be worn for long periods to avoid relapse, representing a challenge for patients, particularly when removable retainers are used, as these require ongoing compliance.

Compliance is one of the most researched areas within all disciplines of healthcare but is still one of the least understood (Thummak et al., 2023). Compliance within dentistry and orthodontics is not investigated to the same extent but the same issues are raised. In orthodontics, compliance mostly relates to keeping appointments, maintaining good oral hygiene and wearing removable appliances (Aljabaa et al., 2015).

Although compliance with removable retention appliances is essential for successful post-treatment stability, there is a lack of knowledge surrounding why some patients comply and others do not. Satisfaction with treatment outcomes and perceptions of different retention strategies have been analysed, using self-reported questionnaires (Forde et al., 2018; Krämer et al., 2021). A randomised controlled trial, published in 2023, showed a high degree of treatment outcome satisfaction after 5 years of retention (Krämer et al., 2023). This trial also revealed that self-reported wear time after 5 years of retention with a VFR, with a recommended wear time of at least 1–2 nights per week, had decreased considerably. Only 28% of the patients reported that they still wore their VFR as recommended, compared with 90% of patients after 18 months of retention.

It is difficult to capture all the aspects of patients’ perceptions and compliance using self-reported questionnaires. A qualitative research approach offers the possibility of gaining deeper insights into patients’ experiences and facilitates the identification of factors that influence compliance. Qualitative studies in orthodontics, specifically concerning removable appliances and compliance, are few in comparison to the total number but have increased in recent years. Some qualitative studies concerning compliance with functional appliances (El-Huni et al., 2019; Kettle et al., 2020) and compliance with retention appliances (Al-Moghrabi et al., 2019; Frawley et al., 2022; Smorthit et al., 2025; Wilson et al., 2023) have recently been published, all however, with different analytical techniques. The Grounded Theory (GT) method is designed to construct a theory that offers an abstract understanding of one or more core concerns in the studied field (Charmaz, 2014; Glaser and Strauss, 1967; Strauss and Corbin, 1998). It can include all research steps, from data collection to analysis and, finally, theory construction. The developed theory can help researchers generate new concepts that can be tested and applied in clinical practice. Constant comparisons are central concepts in GT analysis, which means that text analysis and data collection are ongoing simultaneously.

The aims of this study were to enhance knowledge of patient experiences that may affect compliance with removable retention appliances, and to explore their strategies for improving adherence to wear recommendations, using GT methodology. In addition, we aimed to explore aspects that may enhance our knowledge in developing effective retention strategies.

## Methods

### Participants

The participants in this study were strategically recruited, using purposive sampling, from recruits to a randomised controlled trial (RCT) comparing two different long-term retention protocols, conducted at the Orthodontic Clinic, Public Dental Health Service, Region Gävleborg, Gävle, Sweden. Post-treatment stability, measured using digitised plaster models (Krämer et al., 2020, 2023), and patients’ perceptions of the retention phase, assessed by questionnaires (Krämer et al., 2021, 2023), were analysed and compared between a group with removable VRFs in both jaws and a group with a VRF in the maxilla and a bonded cuspid-to-cuspid (CTC) retainer in the mandible.

To ensure the long-term perspective the inclusion criterion for the present interview study was attendance at the 5-year follow-up visit, which included 74 patients. The reason for purposively selecting the invited participants was to ensure a broad range of experiences within the study group. The selection was based on post-treatment dental changes at the 5-year follow-up visit but also ensuring that both sexes and both retention protocols were represented (Krämer et al., 2023). An experienced female orthodontic specialist who was not involved in the interviews made the selection. The study was approved by the Swedish Ethical Review Authority (Dnr. 2020-04191). Standard for reporting qualitative research was followed (O’Brien et al., 2014).

### Data collection

The participants were contacted by phone and invited to participate in the present qualitative study. Before the interviews were conducted, all participants were informed about the study, confidentiality and the possibility to withdraw from the study at any time without giving any reason. After signing a written consent form, audio-recorded face-to-face interviews were conducted in a non-clinical setting at the Orthodontic Clinic by one of the authors (AK), a female orthodontist and PhD-student without any prior experience in qualitative research. Her background knowledge includes a doctoral study course concerning interviews and observations as qualitative data collection methods and continuous coaching within the research team. The interviewer was known to the participants as she had enrolled them to the previous RCT and performed all follow-up examinations during the 5-year period.

A topic guide, based on assessments from previous questionnaires together with professional opinions within the research team and results from other studies, was developed and pre-tested in several pilot interviews before the study started (Al-Moghrabi et al., 2019; Frawley et al., 2022; Krämer et al., 2021, 2023). These pilot interviews were recorded and discussed within the research team, to prepare the interviewer for the task. In addition to AK, the team also included CSM, a female specialist in orofacial pain and jaw function with extensive experience in qualitative research, and IF, with extensive expertise in orthodontics and long-standing skills in quantitative research, especially regarding patients’ perceptions of different phases of orthodontic treatment. Different backgrounds and perspectives were considered an advantage during the analysis.

The initial topic guide covered open-ended questions regarding orthodontic treatment, reasons for treatment to satisfaction with the outcome, experiences of the two current retention protocols and adherence to wear recommendations. The interviews lasted on average 23 min (range 12–37 min) and were ended with the interviewer making a brief summary to ensure consensus. The participants also gave their consent for the interviewer to contact them again in case any uncertainties arose during the analysis process. All interviews were transcribed verbatim and then cross-checked with the audio files for accuracy. During the ongoing data analysis, the topic guide was continuously reviewed and modified in accordance with the GT methodology. Sampling continued until theoretical saturation was reached, meaning that the identified categories were solid, well-developed and consistently recognisable across all interviews and no more concepts, categories or theoretical insights emerged from the continuous data analysis.

### Data analysis

Data were analysed according to GT with a methodological approach closely aligned to Glaser’s classical GT, emphasising the emergence of theory from data through constant comparison, theoretical sensitivity and the avoidance of pre-formulated categories.

The primary goal of GT is to generate new theories, hypotheses and concepts grounded in empirical data; however, the method can also be applied in a more deductive way, to test or refine existing theories (Charmaz, 2014; Glaser and Strauss, 1967; Strauss and Corbin, 1998).

This study is grounded in a relativist ontological position, which is based on the idea that social reality is not fixed but made up of many perspectives. It also adopts an interpretivist–constructivist epistemology, whereby knowledge is collaboratively produced through interactions between researchers and participants. As a research team, we recognised that our different backgrounds and prior assumptions could affect how we collected and analysed the data. To address this, we, throughout the study, had collaborative discussions about our assumptions, compared our interpretations and explored different ways of understanding the material to reach analytic consensus (Charmaz, 2014).

To enhance credibility, we used the investigator triangulation technique (Carter et al., 2014; Dahlgren et al., 2004; Patton, 1999). Our research team worked together continuously and was involved in all stages of this study. The analyses were performed by three researchers (AK, IF, CSM), all of whom have different perspectives, which was beneficial for the process. Data analysis was carried out iteratively during the research process, in line with the principles of constant comparison and theoretical sampling. Memo-writing was also used throughout the research process to document our thoughts, reflections and theoretical ideas related to the data, codes and emerging concepts. These strategies strengthened the credibility and dependability of the analysis by reducing influence of individual researcher bias.

The analysis started with a line-by-line open coding of the transcribed interviews. Initial coding was made separately by going through the texts and first writing down the codes on a concrete level. The codes were close to the text and could even be authentic words that the participants used. The next step was to select which codes were most prominent and cluster them into categories at different levels of abstraction. A category is a central concept in GT and can be described as a concept that organises reality.

Several meetings between the researchers followed, in which comparisons of the codes and categories, as well as hypothetical ideas were discussed and revised continuously, which is another key process in GT-constant comparisons. Discussions continued until consensus on the categorisations was reached and a core category and theoretical framework were formulated. Table 1 shows an example of this process.

**Table 1.** Example of the analysis process with quotations, codes, subcategories and categories leading to the core category, in integration with the other categories.

Participant	Quotations	Codes	Subcategories	Categories	Core category
8	‘I worked a lot and had my mind on other things. I was on pretty strong medication, so I don’t remember much from that time, . . . I was just trying to cope.’	Work Inattentive Strong medications Try to cope	Health issues Medication Age Personality Work and living situations	Individual factors	Pragmatic actions for improved adherence
1	‘. . . you can maybe check a little bit about what they are like as a person, if they tend to forget things and what they need themselves to remember important things like this. I moved around a lot; I had a partner. . . and my parents were divorced, and it was difficult to remember to bring everything.’	Like as a person Forgetfulness Living at different places	–	–	
14	‘Most of us are so young when we have braces that we become a little careless, don’t think any further ahead, and then when 10 years have passed, we regret it. . . and you correct your teeth again in adulthood because they are not completely perfect.’	Young Careless Regrets Retreatment	–		
13	‘. . . and I had a mother who reminded me, she remembered it. Well, until I got a real job. . . because then she felt that now I’m old enough, . . . so that was probably about the time I stopped, because then I didn’t have someone to remind me.’	Mother reminded Old enough Grown-up Mother ceased to remind	Family Background Social support Professional support	Support	
3	‘. . . something that could have helped me is to come in for check-ups to make sure it really fits. If you could come in every 2 or 3 months, or every 6 months. Phone-calls and emails from you can also help.’	Regular check-ups Phone calls Emails Reminders	–	–	
6	‘Well, in the beginning I was very careful, not to let them go back, . . . wanted to keep a good result, . . . now it’s part of the routine too and I think, . . . it’s not a problem to use it so I just do it, just to be safe.’	Careful in the beginning Maintain result Routines Just to be safe	Maintaining satisfying treatment results Logistic routines Digital reminders	Self-motivation and Routines	
4	‘. . . I had it by the bedside table, so I could push it in when I went to bed, when you woke up in the morning, remembering to clean it, and then putting it back. . .’	Bedside table Push it in Wake up Clean it	–	–	

## Results

In total, 15 purposively selected participants (seven men, eight women; mean age = 27 years) took part in the interviews. During the recruitment phase, another 15 presumptive participants were contacted but nine of them had moved from the region/country, three declined to participate and three did not show up at the agreed appointment. The average time span from debond to the interviews was 9.9 years (range = 6–12 years). Four of the participants still wore their VFRs as prescribed by the orthodontist, whereas 11 participants had ceased to comply and reported a wear time of between 1 month and 7 years. The characteristics of the participants, including the values for anterior crowding (Little et al., 1988) at the follow-up appointment 5 years after debond are presented in Table 2.

**Table 2.** Participant characteristics including, sex, age, type of retention, time in retention, self-reported adherence to VFR wear and anterior crowding measured at the 5-year follow-up after debond.

Participant	SEX	Age (years)	Type of retention	Time span after debond (years)	Self-reported wear time	Measurements after 5 years in retention	
						LII maxilla (mm)	LII mandible (mm)
1	F	26	VFR/CTC	12	7 years	1.1	3.21
2	F	32	VFR/CTC	12	1 month	1.86	1.82
3	M	30	VFR/CTC	12	Still	2.52	3.28
4	F	30	VFR/CTC	12	5 years	1.56	0.89
5	M	28	VFR/VFR	12	2 years	1.64	3.58
6	F	29	VFR/CTC	11	Still	1.08	0.75
7	M	27	VFR/VFR	10	2 years	1.77	3.84
8	F	27	VFR/CTC	10	2.5 years	1.77	1.63
9	M	25	VFR/CTC	10	7 years	1.01	2.60
10	F	25	VFR/VFR	8	1 year	1.26	2.97
11	F	28	VFR/VFR	9	Still	1.99	0.71
12	F	24	VFR/CTC	9	Still	2.42	2.33
13	M	25	VFR/VFR	8	2 months	4.39	2.63
14	M	26	VFR/CTC	7	6 years	0.93	3.87
15	M	25	VFR/CTC	6	1 year	0.97	3.14

CTC, cuspid-to-cuspid retainer; F, female; LII, Little’s Irregularity Index; M, male; VFR, vacuum-formed retainer.

**Figure 1.**
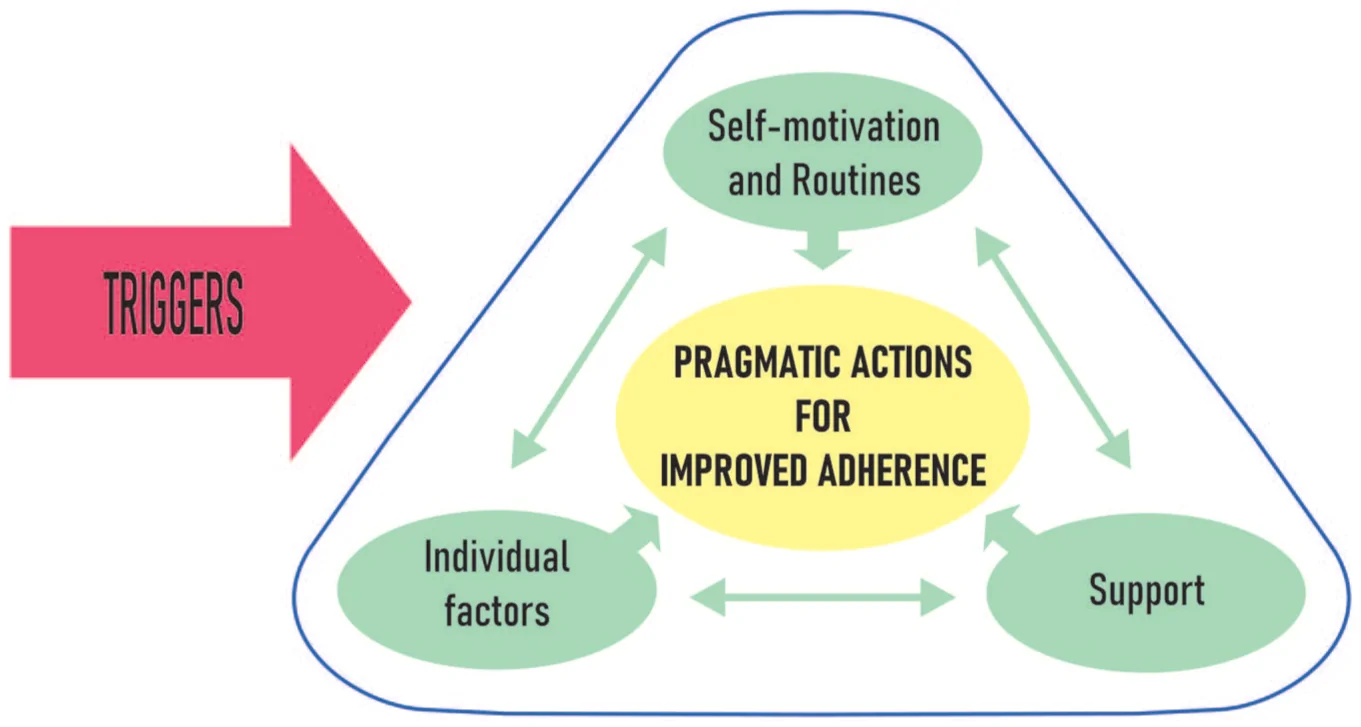
Illustration of the core category with the three interacting categories affecting adherence to wear recommendations with removable retention appliances.

During the analysis process, a core category was identified and labelled ‘Pragmatic actions for improved adherence’, which summarises how orthodontic patients experience compliance with removable retainers and what their strategies to enhance compliance are. The core category was associated with three interacting categories that affected adherence to wear recommendations: Individual factors, Support, and Self-motivation and Routines. The interviews also revealed that reduced adherence to wear recommendations for removable retainers was preceded by a trigger, such as pain and discomfort, impaired quality of life and changed living situation. Figure 1 illustrates the process of managing triggers and enhancing adherence to wear recommendations.

### Pragmatic actions for improved adherence

The core category indicated that the decision process to discontinue complying or to continue wearing the appliance was based on pragmatic patient-centred considerations. Pain after forgetting to wear the retainer early after debond could result in a decision to quit wearing the retainer. Pain and discomfort were consequently most frequently mentioned at the beginning of the retention phase but were also present after a few days of forgetfulness or when wear time was reduced according to recommendations from the orthodontist. Disturbed sleep, constant effort to remember the removable retainer and making sleepovers uncomfortable were issues that the participants described as impairing their quality of life. Lost retainers and potential costs for new ones were other examples that could be a trigger for reduced adherence. During the treatment phase, most of the participants were still in school and lived at home. In the retention phase, however, their living situations often changed due to factors such as studying away from home and military service. These changes made compliance with retention appliances even more challenging.

‘*. . . when I didn’t use it for a few days, it didn’t fit anymore . . . so it hurt. And then it hurt when I tried to sleep, and then it hurt when I took it out the next day, so I felt it was just in the way instead, so I stopped using it.*’ (Participant 5, male, 28 years)

‘*. . . then I lost it, and then I moved, and that was the end of it.*’ (Participant 9, male, 25 years)

The core category also pointed out the responsibility of the profession to facilitate compliance and to provide effective individual retention recommendations.

‘*. . . yes, I was more careful in the beginning. . . but when I hadn’t been here on regular visits for a while, I started to forget to wear it. . . so regular check-ups are important, because then you try it on me. Now, I haven’t been here for a long time. . . so it probably won’t fit any longer.*’ (Participant 1, female, 26 years)

‘*I think. . . you might notice a patient who often reschedules or cancels and. . . or comes late. . . well, now I’m a bit of a time optimist myself and came in last minute, but I came here anyway. But someone who maybe comes 5 minutes late every time, and then I don’t know, it might be possible to see in other ways if they might be a little careless with hygiene or something like that.*’ (Participant 12, female, 24 years)

### Individual factors

This category is broad and describes different individual factors among the participants, such as health issues, age, personality traits, work and living situations, that could affect their ability to adhere to retention protocols. The common denominator for all these individual factors is that they are more or less fixed and thus difficult to influence. However, in interaction with the other decisive categories, compliance still can be enhanced.

Health issues, such as diseases, medications and/or neuropsychiatric diagnoses, can contribute to difficulties in following wear recommendations.

‘*. . . I have ADHD, so for me, this becomes an extra step; I mustn’t forget, and I can get a bit stressed if I do. But I think that for a person who has problems. . . it might just be an extra step in their daily routine.*’ (Participant 11, female, 28 years)

Age and personality traits were also identified as important individual factors that influenced compliance. Terms such as ‘laziness’ and ‘forgetfulness’ were mentioned in connection with reduced compliance:

‘*I think that when you’re younger, you might be a bit careless and perhaps focus a lot on appearance and such. . . you might think, “oh, it doesn’t matter”. But as you get older, you might realise that if you don’t use it, you may need braces again, or the teeth might shift back and look bad again, leading to the whole process all over again.*’ (Participant 6, female, 29 years)

‘*Yes, I’m quite lazy, I’m pretty comfortable, and I don’t really like doing things. . . unless I have to. That’s my personality, and I think that if you feel like I do and are a bit like that, it’s easy to forget things like this.*’ (Participant 8, female, 27 years)

Work and living situation as an adult and still recommended to wear a removable retainer has been described by several participants as a problematic combination. Different working hours, shift work or having to work late at night were also mentioned as obstacles to compliance later in the retention phase:

‘*For me, I don’t want anything in my mouth when I’m asleep. If I’m awake, okay fine. But. . . if you work like I do in a kitchen, it is kind of a bit problematic.*’ (Participant 3, male, 30 years)

### Support

This category encompassed both family support and professional support or the lack of such support. Family support was especially important for younger patients, with the mother described as the crucial person. Siblings and peers assisted with information from their own experiences of the procedures, although they seemed to be less important as support. Overall, the participants expressed a high level of trust in their treating orthodontist; therefore, professional support was considered valuable. When the patients were referred from the orthodontic clinic, a lack of professional support was mentioned as particularly troubling. The participants expressed desires for clearer individual information regarding wear time, more frequent follow-up visits and digital reminders, particularly at the beginning of the retention phase. None of the 15 participants had sought information online.

‘*Maybe something like. . . a call from the clinic. . . checking up if you still use the splint. . . or perhaps a mail or an SMS as a reminder. . . it would have been helpful.*’ (Participant 1, female, 26 years)

‘*At the orthodontic clinic, there was always a proper follow-up every time. . . but now when I’m back at my regular clinic. . . I can’t remember them checking it at any time. . . and I would have appreciated it. I have done so much with my teeth and been so careful all these years with my braces, splints and all that. . . I wish that they took it more seriously.*’ (Participant 8, female, 27 years)

### Self-motivation and Routines

At the beginning of the retention phase, the participants were highly motivated to use the removable retainers (VFRs) according to the recommendation. They were eager to maintain treatment outcome, which they in general expressed as very satisfactory. Four of the participants still wore their removable retainers at the time of the interview, as it was important for them to maintain a satisfactory treatment outcome. Additional costs to replace a lost retainer or the obvious risk for expensive re-treatment were also mentioned as motivators. Time and resources had been invested, by the individual as well as by the professional, so duty in relation to the profession was also an aspect that affected motivation.

‘*. . . as I said, I want to maintain a result, because it’s still a process you’ve gone through and. . . it would be sad if. . . if I stopped using it and it went back. . . but, you have also invested time and resources, even if it’s mostly just me. I was happy with the result and want to keep it.*’ (Participant 12, female, 24 years)

‘*. . . I don’t know. . . just, put them in there in the evening and. . . of course, you don’t feel anything when you sleep. . . but, I don’t know. . . I just couldn’t take it anymore.*’ (Participant 10, female, 25 years)

Self-motivation was also an important factor when the participants had made an independent decision to reduce wear time. In general, the decision was characterised by awareness of the risk of relapse, but also in terms of personal responsibility.

‘*It felt like it wasn’t needed anymore, because, as I said, I gradually reduced to wear it. . . at first I had it every other day, and then a little less,. . . in the beginning you noticed that it was necessary. . . it was sometimes like it didn’t even really fit if I missed 2 days. . . that it needed to tighten again, but after a while it felt like. . . well, even though I. . . when I hadn’t worn it for 3–4 days so. . . it felt like it was ok.*’ (Participant 7, male, 27 years)

Wearing a VFR has been described as requiring constant effort, as it is something that you always have to remember. Good routines were, therefore, described as essential and the participants expressed this in terms of storing the VFR near the bed and having different digital reminders. Some participants also suggested having a second VFR, to make it easier when living at two locations.

‘. . . *if you make a small routine for yourself,. . . every time you go to bed, for example, you have to put them on. . . or, if you set an alarm or reminder in your mobile phone, that can too be something. . . just something to make you remember, because it was. . . it was easy to forget, at least in the beginning, until you had got used to it.*’ (Participant 7, male, 27 years)

Out of the 15 participants, 10 had a VFR in the maxilla and a bonded retainer in the mandible and, therefore, were able to make comparisons. Most of the participants preferred the fixed retainer because:

‘*. . . you don’t have to think about it. It’s there without you having to do anything.*’ (Participant 2 , female, 32 years)

However, some associated strong benefits with the VFRs and would, if the option was available, choose the splint:

‘*. . . oh, maybe I should choose the splint then. . . I’m not bothered by the wire, but it’s there all the time, so sometimes you sit and fiddle with it with your tongue and such, but. . . Yes, maybe I should choose a splint, I think so, because then you don’t need to wear it during the day all the time. . . so a little better. . . and you can eat an apple undisturbed.*’ (Participant 6, female, 29 years)

## Discussion

The participants in this study had all undergone orthodontic treatment in both the maxilla and mandible, followed by a recommendation of long-term retention. The interviews took place approximately 10 years (mean = 9.9 years) after debond, at which time the participants were all adults with completely different ways of life compared to their former selves as teenagers with braces. Despite this, the participants had clear memories of their treatment and retention phases and could even give detailed descriptions of the day when the braces were removed. Although some parts probably were forgotten, this long interval between treatment and interview was considered important and beneficial for an overall understanding of the participants’ perceptions and the concept of adherence. This made this study unique and strengthens our belief that orthodontic treatment is for life. Thus, comparisons with other studies with shorter follow-up periods become more difficult to make.

In the referred literature, both compliance and adherence are used interchangeably to describe the same concept. In the present study, the concepts of compliance and adherence are interpreted as follows: compliance refers to passive obedience to prescribed instructions, whereas adherence denotes active and collaborative engagement in treatment related decisions and behaviours (Tasaduq, 2023).

### Summary of findings

The results from the interviews reveal that failing compliance is always preceded by a trigger, which can occur after a few weeks or after several years. Pain and discomfort and impaired quality of life were present both at the beginning of the retention phase and later when reduced wear time was recommended. Although inconveniences are significantly less severe during the retention phase compared to the active treatment stage, the participants expressed a low tolerance for pain after 1–2 years of retention, which was also found in previously published questionnaire-based studies (Krämer et al., 2021; Wong and Freer, 2005). Changed living situation as a trigger factor is, of course, more important later in the retention phase, although it can already be relevant at the age of 18 years, a common age when many of our patients leave home and go to university or start military service.

‘Pragmatic actions for improved adherence’ is the core category that emerged during the analysis, which indicates that the process to quit or to continue wearing the retainers is governed by practicalities. The three interacting categories leading to the core category are decisive in terms of how the triggers are managed and, as such, can be considered decisive for compliance. These three categories are the basis for the development of strategies for improving adherence to wear recommendations, from both the patient’s and clinician’s perspectives.

Individual factors is one of the interactive categories. This is a broad term that includes age, personality, work and living conditions, and health issues. The results from two previous qualitative studies (El-Huni et al., 2019; Trulsson et al., 2004) have emphasised that parental involvement is necessary for younger children to be compliant with removable functional appliances. The same conditions are probably also true for removable retainers, so age is definitely a parameter to consider when retention is planned. To gain insight into the patients’ personality and general health, to enhance compliance, requires knowledge of the patient’s medical history and effective communication with the patient and parent(s) before tailoring individual recommendations. The participants also suggested that the profession should pay closer attention to patients with frequent late arrivals or non-appearances at scheduled appointments and consider this as a risk factor for weak compliance. A recently published systematic review also stated that personality traits have an impact on pain perception, attitude, satisfaction and compliance in orthodontics (Nicita et al., 2025).

Support is another category that is decisive for compliance. As mentioned above, parental support is essential for patients at a young age, while family support, and especially from the mothers, is important in the long term. Therefore, encouraging parents to continue their support into the advanced teenage years can be of major importance. In agreement with other studies, the participants expressed requests regarding professional support in terms of more-frequent follow-ups and for a longer period, digital reminders, an extra VFR and clear information on how to get in contact in case of problems (Al-Moghrabi et al., 2019; Frawley et al., 2022; Wilson et al., 2023). The latter is especially crucial when a patient is referred back to the general practitioner. Detailed online information in a website from the patient’s current orthodontic clinic could be helpful in solving problems that might arise. None of the participants in this study had sought information online, which was different from other recently published studies (Al-Moghrabi et al., 2019; Frawley et al., 2022).

Self-motivation and Routines is the third interactive category leading to the core. The participants were still very satisfied with their treatment outcomes although they were aware of post-treatment changes in both the maxilla and mandible. None of them, however, expressed any interest in re-treatment. This was also in accordance with the results from the previous 5-year follow-up (Krämer et al., 2023) as well as in agreement with the results from other studies (Karsli et al., 2023). Four participants still wore their removable retainers after 10 years of retention, and the main motivating factor was that they were eager to maintain a satisfactory outcome. To ensure motivation, routines around the retention appliances, such as storage and reminders, are helpful. A recently published qualitative article emphasised understanding of the individual and good routines as important factors for orthodontic retainer adherence (Smorthit et al., 2025). Although their approach with photos taken by the participants as basis for the interviews differs significantly from ours, the results are quite similar, which increases credibility to our topic guide.

According to results from the 5-year follow-up, 28% of the participants still followed the wear recommendations from the professional (Krämer et al., 2023). At the time of the interviews, 4/15 (27%) participants still wore their retainers as recommended. This might indicate that strengthening compliance up to 5 years can facilitate long-term retention.

### Strengths and limitations

Participant selection was based on long-term stability while also ensuring representation of both sexes and both retention protocols. A broad range of participant characteristics enhanced credibility and maximised diversity of experiences within the study group. Analyses were conducted by three researchers with differing perspectives who collectively possessed extensive clinical and odontological research expertise. Preliminary findings were also discussed with colleagues not directly involved in the study. This process of ‘peer debriefing’ strengthened reflexivity by encouraging us, the researchers, to critically reflect on our own positionality within the research process and by incorporating alternative perspectives and constructive critical feedback from individuals outside the immediate study context. Together with investigator triangulation, used as a methodological strategy to enhance credibility, these approaches aimed to deepen the interpretation of the findings. This study was built on the philosophical foundations of GT, adopting a relativist view of reality and an interpretivist–constructivist approach to knowledge. These assumptions guided the use of constant comparison, memo-writing, theoretical sampling and collaborative coding. Together, these iterative practices enabled our research team to critically reflect on emerging ideas and allowed theoretical insights to develop directly from the data rather than from predefined hypotheses. These procedures were directly linked to strategies for enhancing trustworthiness, thereby strengthening the study’s credibility and dependability.

A possible limitation of this study is that all interviews were performed by one orthodontist. Professional attitudes and preconceptions could have influenced the interviews and the analytical process, leading to new theories. That also applies for the analysing team as all three worked in odontology. Another limitation is that the participants were not given the opportunity to comment on the final results. However, given the extended time frame, it was not feasible to contact them again. The interviewer also performed all the examinations at the follow-up visits during the 5-year period, so she was well known to the participants, which can potentially be a limitation. However, this long-term engagement can also be regarded as an advantage: the participants placed great trust in the interviewer because she had been their caregiver for a long time. This may also increase the level of credibility (Dahlgren et al., 2004).

### Generalisability/transferability

To gain an in-depth understanding of the concept of compliance related to long-term retention, qualitative research methods can be valuable. GT was chosen in the present study because it is well established and often used to study complex phenomena in the health sector. To the best of our knowledge, there is no published qualitative research on adherence to different retention protocols using GT as the research method. Although we have established similar results as other qualitative studies addressing the same topic, generalisation is difficult, since different methodologies are used (Al-Moghrabi et al., 2019; Frawley et al., 2022; Smorthit et al., 2025; Wilson et al., 2023). Participants in these four studies also had significantly shorter observation periods compared to our study.

The follow-up time after debonding in the RCT that preceded the interview study was at least 5 years, which differs from the usual clinical routine where patients are referred back to their general practitioner after 1–2 years. This discrepancy and the fact that the participants in these interviews belonged to a group of patients who completed a longitudinal study makes transferability to other settings challenging; this is a topic for further research. On the other hand, one could argue that the criterion of transferability is almost inherently satisfied due to the nature of the GT method. The search for similarities is central to the process of constant comparisons, and the resulting theory/hypothesis is designed to fit the data and to ensure applicability across diverse contexts.

### Interpretation

Clinical implications, based on the results of the analyses in this study, are as follows:

- Thorough communication regarding individual factors, including current life situation with patient and parent(s), before tailoring individual retention recommendations.
- Prolonged support activities from the profession, including distinct individual information together with more frequent follow-up visits and reminders.
- An extra retainer, free of charge, when living in two places.
- Encourage continued family support, which is equally important as professional support.
- Information on how to get in contact with the profession in case of problems. This is especially important when the patients are referred back to the general practitioner.
- Good routines are considered necessary, since wearing removable retainers is described as entailing constant effort on the part of the patient. Recommendations from the profession on useful routines right from the very beginning can be invaluable.

Further research concerning the impact of changed routines on retainer compliance is needed.

## Conclusions

The generated hypothesis is labelled ‘Pragmatic actions for improved adherence’, which proposes that the decision process to cease or to continue wearing a retainer is governed by practicalities. Three interacting predictors—Individual factors, Support, and Self-motivation and Routines—are identified and form the basis for the development of strategies for improving adherence to wear recommendations, from both the patient and professional perspectives. To be successful, strategies to enhance compliance with removable retainers must originate from the individual patient and their life situation. Long-term support from both family and professionals as well as good routines are considered crucial for this process.
